# Utilization of Home and Community-Based Resources by Family Caregivers in a Native American Community

**DOI:** 10.1093/geroni/igab046.1370

**Published:** 2021-12-17

**Authors:** Mary Wyman, Debra Miller, Sunshine Wheelock, Florence Petri, Elijah Metoxen, Nickolas Lambrou, Carey Gleason

**Affiliations:** 1 Madison VAMC/UNIVERSITY OF WISCONSIN, Madison, Wisconsin, United States; 2 Oneida Comprehensive Health Division, Oneida, Wisconsin, United States; 3 Oneida Alzheimer's Community Advisory Board, Oneida, Wisconsin, United States; 4 Oneida Aging & Disability Services, Oneida, Wisconsin, United States; 5 University of Wisconsin, Madison, Madison, Wisconsin, United States; 6 University of Wisconsin, Madison, Wisconsin, United States

## Abstract

Family caregiving is uniquely significant for elder care within American Indian/Alaska Native (AI/AN) communities. Compared to other populations, AI/AN older adults are disproportionately impacted by chronic conditions and AI/AN are more likely to be family caregivers. However, AI/AN are underrepresented in aging research. We describe a successful research partnership with the Oneida Nation of Wisconsin and report results of a recent survey of tribal members and affiliates (N=405), covering demographics of caregiving, awareness and use of home and community-based resources, and perceptions of factors impacting service use. Approximately 42% of respondents were current caregivers; of these, roughly one-third knew how to access various resources. Most common sources of knowledge were a health care/social worker or finding information on their own. Traditional cultural values were viewed as variably supportive of resource utilization, depending on service type. Implications for efforts to address disparities for AI/AN aging and support caregivers will be discussed.

